# Non-invasive intracranial pressure monitoring for high-grade gliomas patients treated with radiotherapy: results of the GMaPIC trial

**DOI:** 10.3389/fonc.2024.1302977

**Published:** 2024-06-11

**Authors:** Mélanie Casile, Emilie Thivat, Fabrice Giraudet, Angeline Ginzac, Ioana Molnar, Julian Biau, Julien Brehant, Blandine Lourenco, Paul Avan, Xavier Durando

**Affiliations:** ^1^ INSERM U1240 IMoST, University of Clermont Auvergne, Clermont-Ferrand, France; ^2^ UMR 501, Clinical Investigation Centre, Clermont-Ferrand, France; ^3^ Clinical Research and Innovation Department, Centre Jean Perrin, Clermont-Ferrand, France; ^4^ INSERM 1107, University of Clermont Auvergne, Clermont-Ferrand, France; ^5^ Radiation Oncology Department, Centre Jean Perrin, Clermont-Ferrand, France; ^6^ Radiology Department, Centre Jean Perrin, Clermont-Ferrand, France; ^7^ Oncology Department, Centre Jean Perrin, Clermont-Ferrand, France

**Keywords:** otoacoustic emissions, glioma, intracranial pressure, monitoring tool, non-invasive, brain tumor

## Abstract

**Introduction:**

Patients with high-grade gliomas are at risk of developing increased intracranial hypertension (ICHT) in relation to the increase in volume of their tumor. ICP change cannot be measured by invasive method but can be estimated by using routine clinical signs, in combination with a standard imaging method, magnetic resonance imaging (MRI). A non-invasive monitoring of ICP could be of interest in high-grade glioma, in particular after radiotherapy treatment with as major side effect a cerebral oedema.

**Patients and Methods:**

This prospective clinical study aimed to compare the ICP changes (estimated by a non-invasive method based upon distortion product otoacoustic emissions (DPOAE) monitoring) with volume changes observed on MRI in patients with high-grade gliomas treated with radiotherapy. DPOAE measurements were performed one month after the end of radiotherapy and then every 3 months for one year. At each visit, the patient also underwent MRI as well as an evaluation of clinical signs.

**Results:**

The variation in the estimate of intracranial pressure readout measured at each follow-up visit (in absolute value with respect to the baseline measurements) was significantly associated with the variation of T2/FLAIR volume (n=125; p<0.001) with a cut off value of change ICP readout of 40.2 degrees (e.i. an estimated change of 16 mm Hg).

**Discussion:**

The GMaPIC trial confirm the hypothesis that the ICP change estimated by DPOAEs measurement using a non-invasive medical device is correlated with the change of the tumor or edema in high grade glioma after radiotherapy. The device could thus become an easy-to-use and non-invasive intracranial pressure monitoring tool for these patients.

**Clinical Trial Registration:**

Clinicaltrials.gov, identifier (NCT02520492)

## 1 Introduction

High-grade gliomas account for the great majority primary malignant brain tumors in adults (1). Radiotherapy is, with surgery, one of the mainstay treatment of these tumors. Intracranial hypertension (ICHT) is one of the major issue for the management of patients with high-grade gliomas.

Indeed, the cranium contains the brain, vessels and cerebrospinal fluid (CSF) in an inextensible bony cavity. The pressure inside this cavity i.e. intracranial pressure (ICP) is relative to these different components. In normal conditions, an increase in the volume of one of these components is compensated by a decrease in the volume of one or more of the components (2). The physiological value of ICP is between 5 and 15 mmHg in adults, but its value can oscillate up to 20 mmHg (3). Blood and CSF are the two components whose volume adapts most readily to maintain a normal ICP. However, if their volume cannot decrease enough, then ICP cannot be maintained causing ICHT (4). In the presence of high-grade glioma, the effect of mass specific to the tumor and/or the appearance of edema, in particular due to inflammatory reaction around the tumor, can be the cause of ICHT. It is defined as an ICP value higher than 20 mmHg (5).

The use of direct measurement of ICP by invasive intra-parenchymal sensor or a ventricular shunt (gold standard method) is not an option for clinical practice in the management of high-grade glioma patients (6). Currently, clinicians rely on indirect clinical and imaging signs to evaluate ICHT.

Over the last ten years, non-invasive measurement methods using auditory sensors have been developed to detect variations in ICP by way of sensitive and rapid measurements (7–13). The cochlea, in response to acoustic stimulation, emits sounds called otoacoustic emissions (14). The mechanical movement of the hair cells in the cochlea creates sound vibrations that propagate back from the inner ear to the outer ear. The cochlear aqueduct provides communication between the perilymphatic space of the cochlea and the subarachnoid space containing the CSF. It therefore maintains the balance of ICP with the intracochlear pressure (15). Therefore, any change in ICP will result in a change in intracochlear pressure. This then causes a change in the acoustic impedance of the inner ear which also leads to a change in otoacoustic emissions. The sound vibrations emitted at the outer ear are thus modified with each variation in ICP. Their modifications provide non-invasive readouts of ICP changes, even though the huge inter-individual variability in baseline inner-ear properties precludes absolute ICP to be assessed. This phenomenon has been investigated and validated in three studies using distortion product otoacoustic emissions (DPOAE) (8, 16, 17) enabling the detection of changes as small as 4 mmHg (10).

A DPOAE measurement technique has been developed (ECHODIA^®^, Clermont-Ferrand, France) making it possible to observe isolated or reproducible pressure variations in the cochlea, with the aim of revealing cochlear hydrops or increased ICP (16, 18). The GMaPIC exploratory study was the first-in-man evaluation of this medical device to monitor estimate ICP in patients treated with radiotherapy for a high-grade glioma. The main objective was to compare the estimate variation as detected by a non-invasive method based upon DPOAE monitoring with magnetic resonance imaging (MRI) outcomes.

## 2 Patients and methods

### 2.1 Study design

The GMaPIC study is a prospective, interventional, medical device, non-randomized, single patient group study to test a medical device ECHODIA^®^ in a longitudinal cohort. This study has been registered on Clinicaltrials.gov (NCT02520492), approved by the Committee for the Protection of Persons of Sud-Est VI, and authorized by the National Agency for the Safety of Medicines and Health Products (ANSM) in November 2014.

### 2.2 Patients eligibility

The study population consisted of patients aged 18**–**65 years with histologically confirmed high-grade glioma (according to the 2016 WHO Classification of Tumors of the Central Nervous System) for whom surgery consisted in stereotactic biopsy or incomplete resection and treated with adjuvant radiotherapy. Eligible patients were included at the initiation of radiotherapy. All patients provided written informed consent prior enrollment.

### 2.3 Interventions

**Figure 1:**
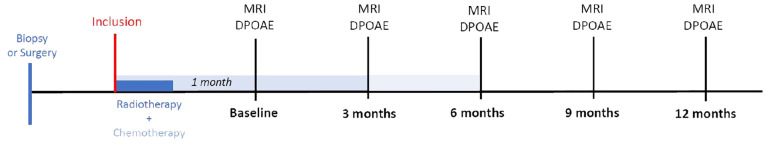
Study intervensions.

Radiotherapy consisted in a total dose of 60Gy using a volumetric modulated arc therapy (VMAT; Rapidarc^®^, Varian Medical Systems, Palo Alto, CA, USA) technique. Contouring was done according to current guidelines (19). Concomitant and adjuvant temozolomide was prescribed according to recommendations (19). One month after the end of radiation therapy and then every 3 months for one year, each patient underwent: DPOAE measurements, MRI, and an evaluation of clinical signs (**Figure 1**).

#### 2.3.1 MRI

MRI included T1, Flair and post-gadolinium T1 sequences. Disease assessment was determined using the response assessment in neuro-oncology (RANO) criteria (20, 21). On each MRI, Flair volumes and post-gadolinium volumes were delineated.

#### 2.3.2 ICP estimation by DPOAE measurements

DPOAE measurements were performed using the ELIOS^®^ device (ECHODIA^®^, Clermont-Ferrand, France). First, an otoscopy was performed to inspect the eardrum and the external auditory canal. If this was well cleared, a tympanometry measure was carried out to check the mobility of the tympanic membrane and middle ear in response to calibrated changes in the air pressure in the ear canal. A normal tympanogram is centered on 0 daPa, i.e. when the pressure on both sides of the tympanum is balanced. Performing this tympanometry ensures that the measurements are not confounded by a change in middle ear pressure.

**Figure 2:**
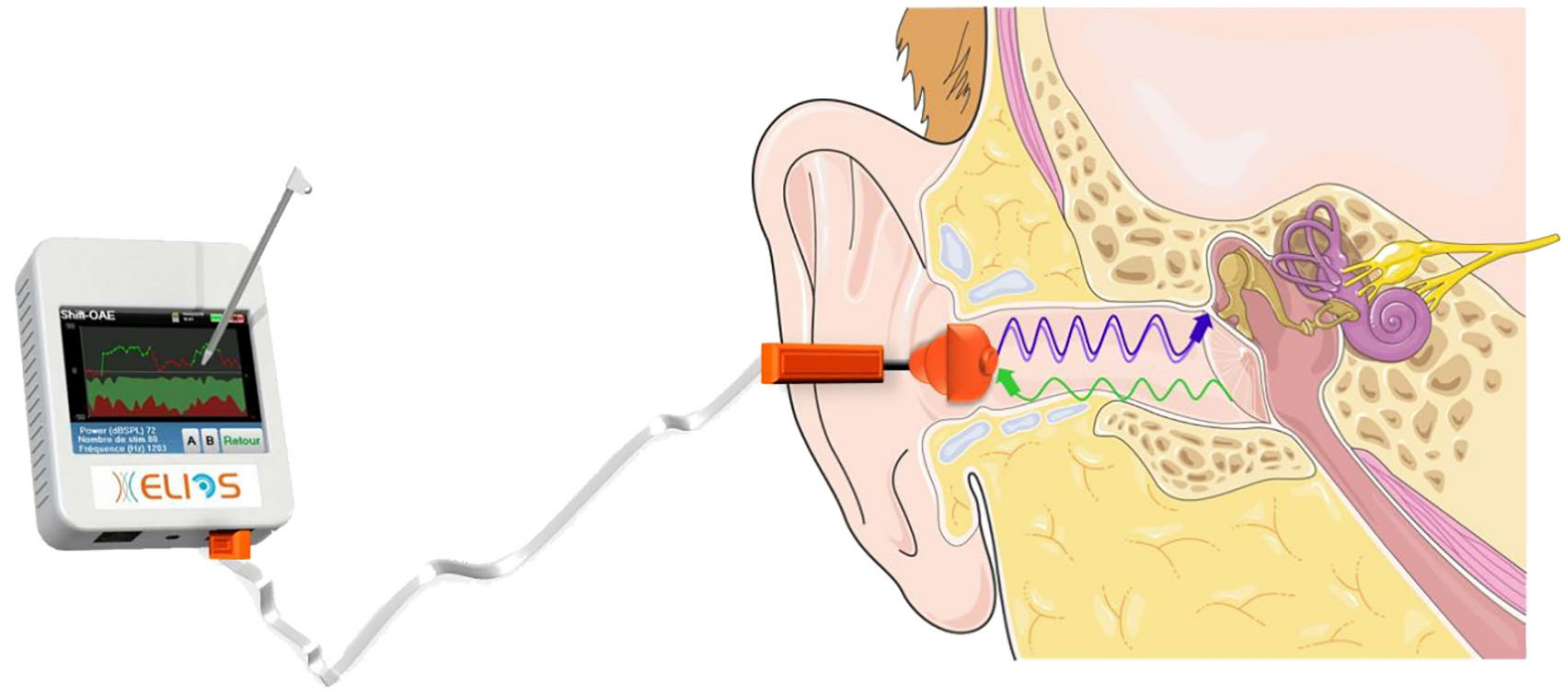
DPOAE measurements performed using the ELIOS® device.

Once these two tests were performed, a sound transmitter was placed in the patient’s external ear canal to send two continuous pure tones to the cochlea. A microphone, placed in the patient’s outer ear, recorded the distortion DPOAE received through the middle ear corresponding to the frequency 2f1-f2 (22). This enabled real-time monitoring of the cochlear pressure, and indirectly the ICP. This device was connected to the ELIOS^®^ device, capable of calculating the phase shift between the emitted and received sound waves, as a function of time, known as shift-OAE (**Figure 2**).

To collect one shift-OAE data point, the ELIOS^®^ device calculates over a few seconds the degree of phase shift between the sound waves emitted and received. The average of 4**–**5 phases data provides a stable readout of shift-OAE (in degrees). Past comparisons between shift-OAE measurements and concomitant invasive ICP measurements in neurosurgery have shown that a change in readout between two measuring sessions of plus or minus 10 degrees relates to a variation in ICP of about 4 mmHg (8). In typical recording sessions, the shift-OAE of a subject at rest in a quiet room remains stable within less than 10 degrees, fluctuations of a few degrees being attributed to acoustic noise, so that a variation of 10 degrees is considered clinically significant.

To ensure the quality of the data, all measurements were examined to determine if they were analyzable. Indeed, in a situation where the earmold has moved during the measurement, the phase average may vary by more than 10 degrees and could be mistakenly considered as a variation of the ICP estimate. In this case, the frequency levels of f1 and f2 will be affected. A difference between f1 and f2 varying by more than 3 decibels during the measurement is considered to be a phase shift. In this situation, the results are unpredictable, so the data obtained are unfortunately not usable (23). Finally, if the ear canal is not sealed against surrounding noise or if the noise is too high during the measurement, the signal of interest will be confused with the background noise. This is considered to be the case when the signal to noise ratio is less than 2 decibels. The results obtained in this situation are also unpredictable. The ECHOSOFT^®^ software connected to the device allows access to this data.

This software also has a functionality that allows an automatic correction, neutralizing the variation of the phase average that can be related to a minor displacement of the earmold. This functionality has been tested and validated to be applied to ELIOS^®^ devices in order to provide more consistent, repeatable and accurate values (23).

### 2.4 Statistical analysis

R software was used for the statistical analysis. Patient characteristics were described using standard distribution parameters: mean and standard deviation or median and range for quantitative parameters and counts and frequencies (%) for categorical parameters.

To investigate the relationship between changes in ICP estimate measures and changes in MRI measurable volumes, or the clinical evaluation of progression, mixed effects linear regression models with subject as random intercept were used to account for the repeated measures design. We consider absolute change with respect to baseline, where the baseline visit is the visit done one month after radiotherapy. Changes in ICP estimate measures are considered in absolute value, since we are only interested in the magnitude of the change, and not its sign. The maximally selected rank statistics with Monte Carlo approximation was used to identify the optimal cutoff value on ICP variation.

Median follow-up was computed using reverse Kaplan-Meier method, with confidence interval based on log-log transformation.

## 3 Results

### 3.1 Patient characteristics

**Figure 3:**
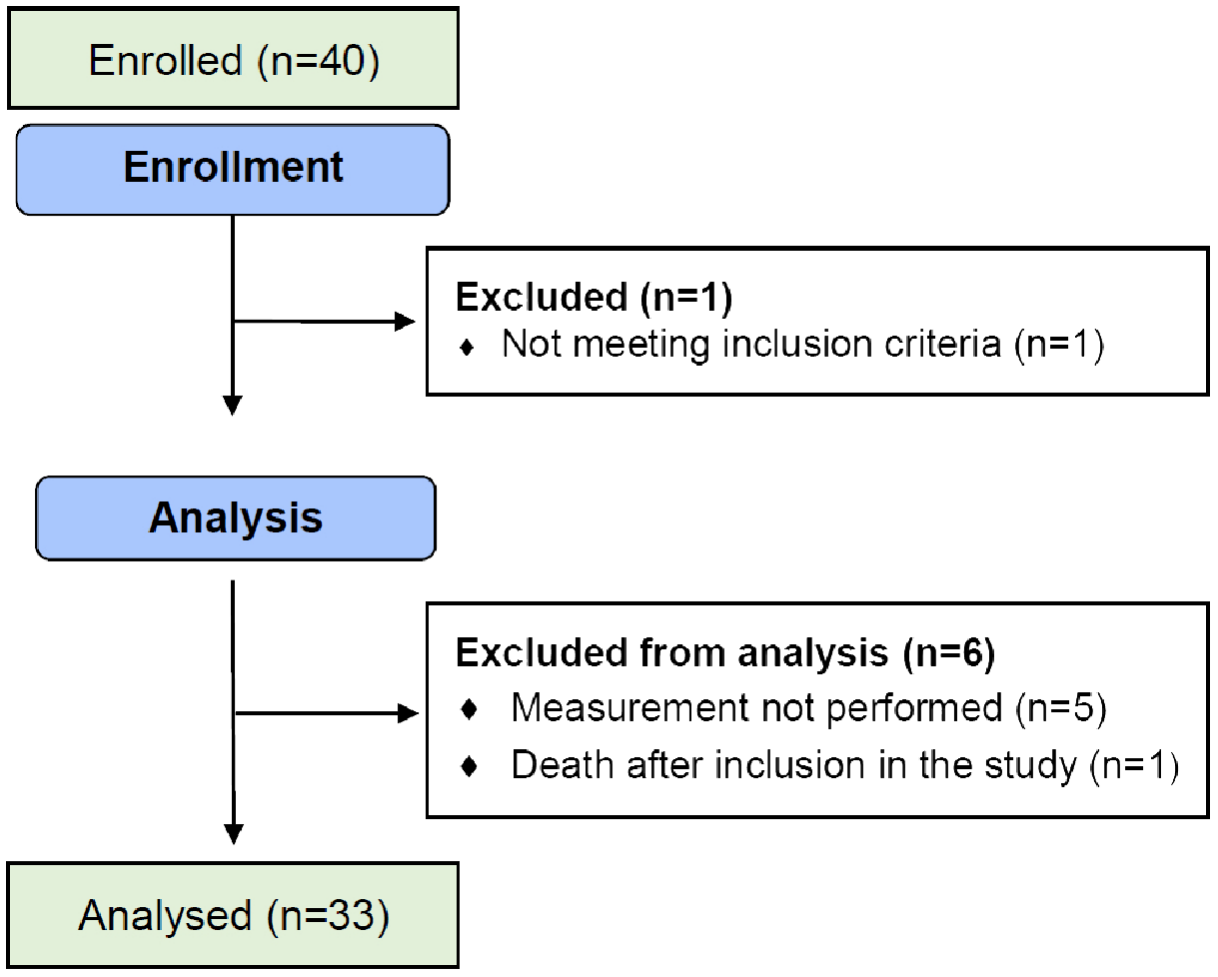
Flow diagram of the progress of the trial.

Forty patients were included from April 2015 to December 2018. One patient was wrongly included, one patient died before the first evaluation, and for 5 patients, the DPOAE measurements collected were not technically exploitable. For one patient, radiotherapy was not technically feasible due to patient noncompliance, and thus the patient only received chemotherapy, but was still kept for the analysis. Thus, 33 patients were considered in this final analysis, and were evaluable for at least 2 evaluations (**Figure 3**).

The patients characteristics are presented in **Table 1**. There were 23 men (69.7%) and 10 women (30.3%). The median age was 49 years (18–64). The histology was as follows: 25 (75.7%) glioblastoma, 6 (18,2%) grade III astrocytoma, and 2 (6.1%) grade III oligodendroglioma. Nineteen patients (57.6%) had partial surgery and fourteen a stereotactic biopsy (42.4%). Twenty-nine patients (90.6%) were treated with temozolomide concomitantly with radiotherapy.

**Table 1:** Patient baseline characteristics.

Baseline characteristics		Values no. (%)
Gender	Female	10 (30.3%)
	Male	23 (69.7%)
Age (years)	Median [min-max]	49 [18–68]
Histology	Grade III Astrocytoma Grade III Oligodendroglioma Glioblastoma	6 (18.2%) 2 (6.1%) 25 (75.7%)
Prior treatment before inclusion	No Yes	31 (93.9%) 2 (6.1%)
Partial Surgery and Biopsy	Partial surgery Biopsy only	19 (57.6%) 14 (42.4%)
Therapeutic management	Radiotherapy only Radiotherapy and chemotherapy	3 (9.1%) 29 (90.6%)
	Chemotherapy only	1 (0.3%)

### 3.2 Treatment outcomes and MRI analysis

The median follow-up was 11.8 months. Thirty-three patients (100%) were evaluable at 3-month follow-up, 30 (91%) patients at 6-month, 27 (82%) patients at 9-month, and 21 (64%) patients at 1-year. At the end of 12 months follow-up, 16 (48.5%) patients remained stable, 11 (33%) patients had progressed and 6 (18%) patients had a partial response, according to the RANO criteria. Most patients (n=18; 55%) progressed at least once during their follow-up.

Mean T1/Gadolinium and T2/Flair volumes were 37.5cc (4.5–114) and 125cc (12–368) respectively at baseline. 52% and 58% of patients had a >10% increased of T1/Gadolinium and T2/Flair volumes respectively during follow-up.

### 3.3 DPOAE measurements

The DPOAE measurements results are presented in **Table 2**. In total, of the 138 visits made during the study, 4 (2.9%) measurements were not performed. In addition, 7 (5.1%) measurements were not analyzed because of a signal-to-noise ratio below 2 decibels due to either a lack of sealing or too much background noise. For another measurement (0.7%), a significant displacement of the earmold during the measurement was identified. Moreover, one last measurement (0,7%) was not analyzed because no imaging was performed on the day of the measurement. The data from these 13 measurements (9.4%), which were not exploitable, were not used for the final analysis. In the end, 90.6% of the data could be analyzed.

**Table 2:** DPOAE measurements.

		Baseline	3 months	6 months	9 months	12 months	All
	DPOAE values (median, IQR)	116.5 (55 – 136)	116 (56 – 133)	122 (74 – 144)	101 (77 – 145)	104 (75 – 149)	115 (64 – 143)
Evolution of DPOAE values	Absolute values (median, IQR)		18.3 (5 – 35)	18 (11 – 35)	16 (10 – 25)	18 (6 – 38)	18 (7 – 36)
	Variation > 10° (n/total n)		18/28	20/27	17/23	7/12	31/33
	Stable (n/total n)		10/28	7/27	6/23	5/12	2/33

All patients but 2 (31/33) had at least 1 shift-OAE of 10 degrees (considered as clinically significant) during their follow-up.

### 3.4 Correlation between DPOAE measurements and MRI analysis

**Figure 4:**
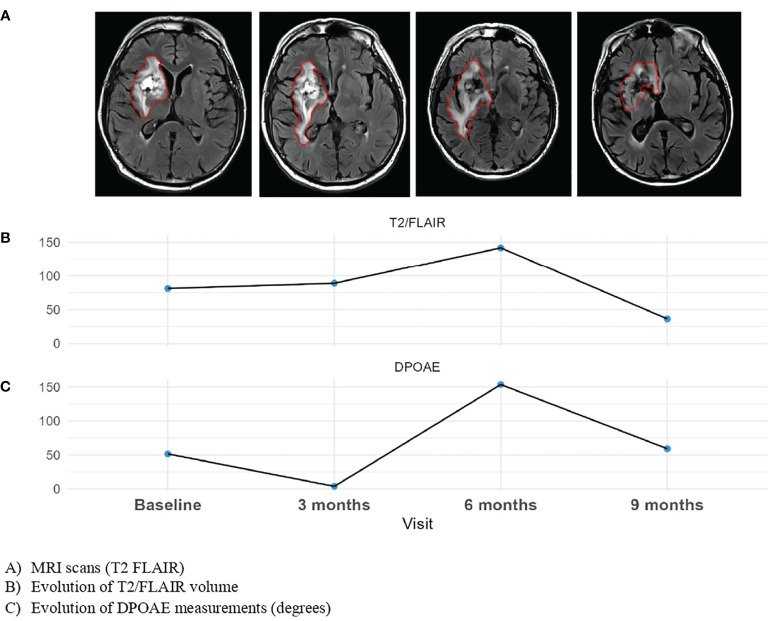
Evolution of DPOAE and MRI evolution to visits of one planet.

**Figure 5:**
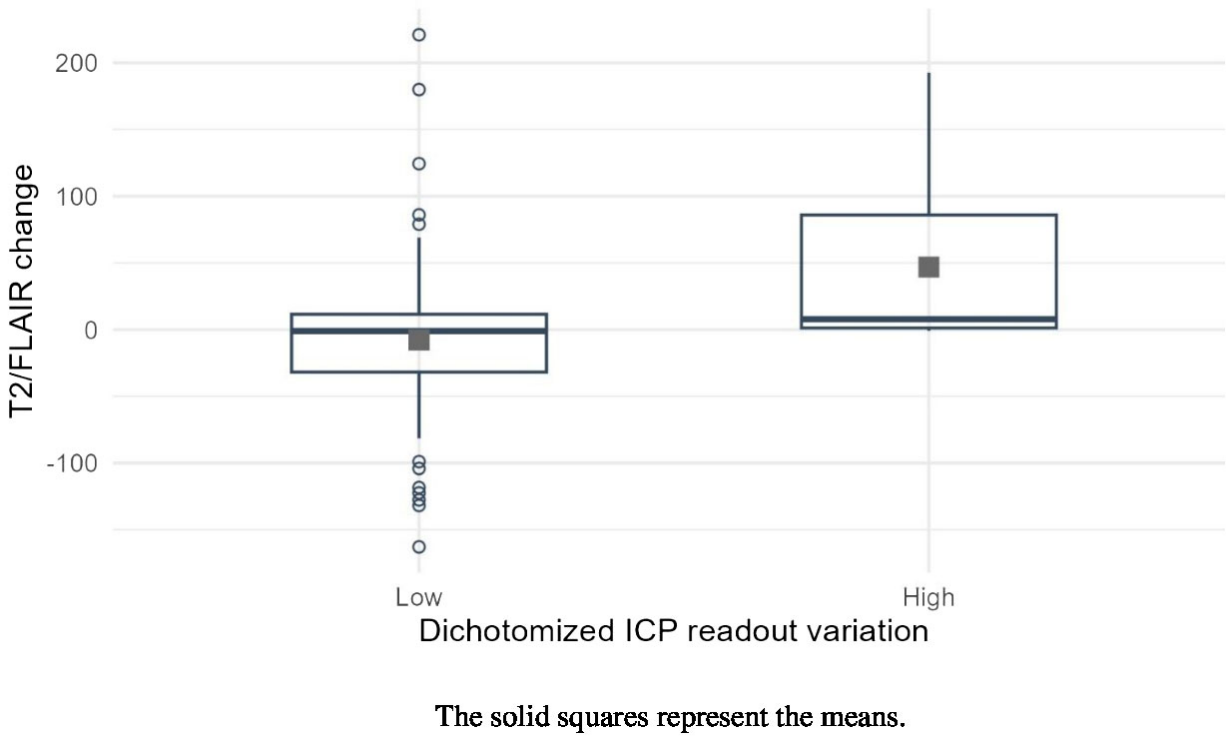
Boxplots of T@/FLAIR volumes according to the dichotomized variation of intracranial pressure estimate.

Change in DPOAE measurements in absolute value with respect to the baseline measurements in the course of the follow-up of the patients was statistically significantly associated with the change of T2/FLAIR volume as dependent variable (estimate of the fixed effect: 1.05, 95%CI (0.51, 1.59), p<0.001, mixed model) (**Figure 4**) and with the change of T1 Gadolinium volume (0.2, p=0.043). The cut-off value on the change in ICP estimate associated with the best separation of values of T2/FLAIR variation into two groups was 40.2 degrees in absolute value (estimate 60.4, 95%CI (30.1, 90.7), p<0.001, multiple comparison-adjusted p-value=0.02) (**Figure 5**). Concerning relationship between the estimate ICP change and the clinical evolution, there was also a statistically significant difference in the change in DPOAE measurements in absolute value and the clinical evaluation of the progression (15, 95%CI (3, 27), p = 0.02).

## 4 Discussion

This study is the first-in-man exploratory study to propose a non-invasive method of monitoring indirectly ICP in patients with high-grade glioma treated with radiotherapy. Indirect ICP measurement used a commercially available audiological device, ELIOS^®^, with DPOAE data-processing software specially designed for providing an ICP-related readout. Interestingly, the relative change in ICP estimate between the initial and current situations in the course of the follow-up of the patients was significantly associated with the increase in the T2/FLAIR volume. We also found a significant correlation with the change in T1Gado volumes, but to a lesser degree. In addition, this study has shown that the majority of patients (93,9%) had a significant change in ICP estimate at some time during their follow-up.

The finding that the change in DPOAE measurements associated with the best separation of values of T2/FLAIR variation into two groups was 40.2 degrees, translates into an ICP difference of 16 mmHg between the compared situations, according to the calibration of the ELIOS^®^ equipment. In the present context, it is the presence of an inflammatory reaction around the tumor that could be related to radiotherapy, as explored by T2/FLAIR imaging, that entails the risk of an increase in ICP. The observed 16 mmHg-limit in ICP change makes sense as in neurosurgery, ICP is considered abnormal above 20 mmHg (5). Thus, it suggests that the measurements performed with the ELIOS^®^ device, calibrated in acute neurosurgical or neurointensive care procedures, remain reliable in the context of long-term follow-up of glioma patients.

The number of inclusions required in the study was knowingly based on the number of patients that could be expected to sign in in the given period in view of the center’s active file. Despite the rather small sample, significant correlations were observed. These results should therefore be validated with a larger-scale study including a control group for evaluating the efficacy of DPOAE measurement method against the current standard of care or against other noninvasive ICP monitoring methods.

Of course, a better standard for ICP measurement would have been the use of an intra-parenchymal sensor or a ventricular shunt (24), but this is only acceptable in an acute context in a neurosurgical environment. Moreover, due to infectious risks, the procedure cannot be repeated along the course of several months as required by glioma follow-up. Some non-invasive ICP methods tested recently are based on the mechanical properties of intracranial structures, and the presence of an evolutive tumor precludes their utilization. Other techniques exploit properties of extra-cranial structures, ultrasonic, vascular, ophthalmic in addition to the current audiological one used in the present study (25, 26). None of the non-audiological techniques shows better accuracy, less limitations or less operator-dependency than the audiological ones (27), whereas the present method turned out to be easily applicable in clinical practice as only 5 patients of 40 had inappropriate ICP estimate data.

The present results suggest that the use of the ELIOS^®^ equipment could complement monitoring of the disease by the usual imaging procedures. Currently, patients undergo a tumor evaluation every 3 months. With this medical device, additional measurements between follow-up visits could be planned. An increase in ICP readout associated to some pattern of clinical symptoms might enable treatment adaptation. In case a large increase would be detected, it might alert the oncologist to possible tumor progression. Overall, non-invasive measurement of ICP estimate with the tested device could improve the management of patients with high-grade brain tumors at lower cost and risk. A detailed cost benefit analysis should be included in larger comparative study.

Before these ambitious goals can be achieved, one must examine whether the system allowing non-invasive measurements of ICP changes is reliable enough. In this study, eight measurements (5.8%) could not be analyzed because of an insufficient signal-to-noise ratio, due either to a poor seal of the measuring probe in the ear canal or to too much background acoustic noise. A non-repeatable positioning of the measuring probe from one session to the next can also yield results that are unreliable. A study conducted at the University Medical Center Groningen on 17 volunteers with no history of hearing, vestibular or neurological disorders tested a modified version of the ELIOS^®^ device (version 2) and demonstrated improved robustness, stability and accuracy of measurements (23). This study proposed the use of foam ear plugs that better fit the ear canal, despite their single size, an advantage since it relieves the operator of having to choose a proper ear plug. Software improvements also provide real-time information on probe positioning and quality of seal (23).This enables the user to be guided in positioning the probe in the same position as for past measurements in the same ear. Overall, this improved version could reduce the number of unusable data and provide better reproducibility, data reliability and device’s usability for further studies.

## 5 Conclusion

The GMaPIC trial demonstrated that the ICP changes for patients with high-grade glioma through the use of a non-invasive medical device based on the measurement of DPOAEs, between the baseline visit and the follow-up visits, were mainly associated with an increase in the T2/FLAIR volume. The device, developed by the company ECHODIA^®^ could thus become an easy-to-use ICP monitoring tool for patients with high-grade glioma, making it possible to adapt treatments and possibly control tumor progression.

## Data availability statement

The data analyzed in this study is subject to the following licenses/restrictions: The datasets presented in this article are not freely available for reasons of privacy. Requests to access the datasets should be addressed to the corresponding author. Requests to access these datasets should be directed to emilie.thivat@clermont.unicancer.fr.

## Ethics statement

The studies involving humans were approved by French Ethics Committee (Comité de protection des personnes Sud-Est 6). The studies were conducted in accordance with the local legislation and institutional requirements. The participants provided their written informed consent to participate in this study.

## Author contributions

MC: Writing – review & editing, Writing – original draft. ET: Writing – review & editing. FG: Writing – review & editing. AG: Writing – review & editing. IM: Writing – review & editing. JBi: Writing – review & editing, Writing – original draft. JBr: Writing – review & editing. BL: Writing – review & editing. PA: Writing – review & editing. XD: Writing – review & editing.
